# Development of a Rotary Ultrasonic Motor with Double-Sided Staggered Teeth

**DOI:** 10.3390/mi12070824

**Published:** 2021-07-14

**Authors:** Xiaohui Yang, Dongdong Zhang, Rujun Song, Chongqiu Yang, Zonggao Mu

**Affiliations:** School of Mechanical Engineering, Shandong University of Technology, Zibo 255000, China; zhangdongaugust@163.com (D.Z.); songrujun@sdut.edu.cn (R.S.); yangcq@sdut.edu.cn (C.Y.); muzonggao@163.com (Z.M.)

**Keywords:** bonded-type, double-sided staggered teeth, traveling wave, ultrasonic motor

## Abstract

Based on the conventional structure of traveling wave ultrasonic motor, a rotary ultrasonic motor with double-sided staggered teeth was proposed. Both sides of the stator could be used to actuate the rotors to rotate and output torque. Moreover, the staggered teeth in the stator could be dedicated to accommodating the piezoelectric ceramic chips. Under the excitation of two alternating voltages with a 90° phase difference, a traveling wave could be generated in the ring-like stator. Then, a rotary motion could be realized by means of the friction between the rotors and the driving teeth of the stator. The finite element method was adopted to analyze the motion trajectories of the driving tips. Moreover, the experimental results showed that the load-free maximum speed and maximum output torque of the prototype were 99 rpm and 0.19 N·m at a voltage of 150 V_p_ with a frequency of 28.25 kHz.

## 1. Introduction

The ultrasonic motor (USM) is a new type of actuator based on the converse piezoelectric effect [1,2,3], which has attracted a lot of attention due to its excellent features, such as being lightweight, its instant reaction, low-noise, higher position accuracy and self-locking [4,5,6,7]. The stator is usually a composite elastomer of metal and piezoelectric ceramics in a special structure configuration. The driving nodes on the frictional contact surfaces of the stator vibrate in an elliptic trajectory, which drives the rotor by the frictional force [8,9,10,11,12].

According to the vibration form of the stator, the USM can be classified into standing wave motors [13,14], traveling wave motors [15,16,17] and composite vibration modes motors [18,19,20]. The standing wave motors usually have merits of simple structure and large output force; its disadvantages are large velocity perturbation and difficulty of bidirectional driving [21,22]. The composite vibration modes motors exhibit high speed and large torque but usually have the problem of frequency degeneration [23]. The traveling wave motors have better control performance than the above two types [24,25,26]. Furthermore, the bonded-type ring motor may be the most typical traveling wave ultrasonic motor, which is widely used in many fields such as camera auto-focus systems and medical instruments [27]. Moreover, generally, the flexural mode of a metal ring is used to excite the axial vibration of the stator. However, one side of the stator in the conventional traveling wave ultrasonic motor is piezoelectric ceramics, another side of which is the driving teeth. In other words, only one side of the stator is used to drive the rotor, while the traveling wave energy on the other side is wasted.

A rotary traveling wave ultrasonic motor with double-sided staggered teeth is proposed in this study. The teeth are staggered on both sides of the stator ring, and every piezoelectric ceramic chip is placed between the two groups of teeth. If two alternating voltages with a 90° phase difference are applied to the top and the bottom ceramic chips, respectively, a traveling wave could be generated in the stator, which can drive the two rotors to rotate in the same direction. If the phase difference of the two alternating voltages is changed to be −90°, the dual-rotor system will be rotated in the reverse direction. In this study, the structure and operating principle of the proposed ultrasonic motor are presented. Then, the vibration characteristics of the stator, including resonant frequencies and motion of the driving nodes, are analyzed by way of the finite element method. Finally, an experimental prototype is assembled and tested.

## 2. Structure of The Motor

**Figure 1:**
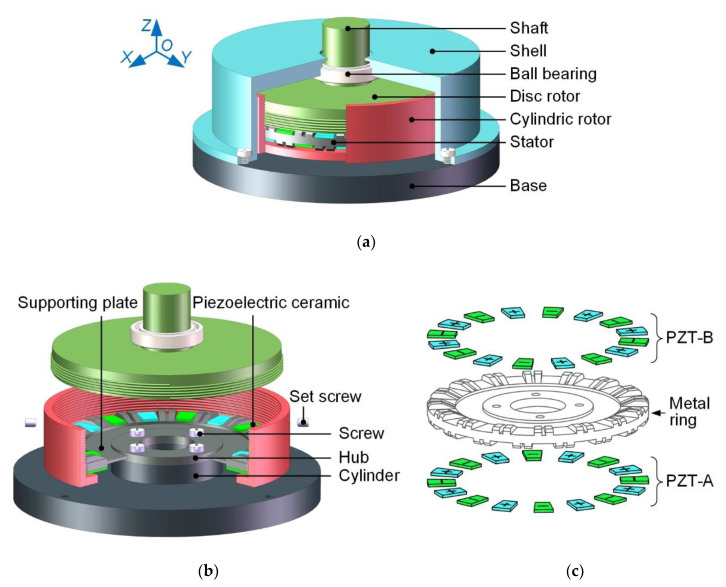
The structure of the proposed motor: (**a**) assembly view; (**b**) internal structure view; (**c**) explosive view of the stator.

Figure 1 shows the overall structure of the proposed motor, which is composed of five main parts: the stator, the dual-rotor system, the ball bearing, the base and the shell.

In Figure 1a, the dual-rotor system mainly comprises the disc rotor, the cylindric rotor and the shaft. The shaft and disc rotor are integrated to realize the mechanical output. The disc rotor and the cylindric rotor are connected with each other through the thread, which can adjust the pre-pressure applied on the driving teeth. Two screw holes are set on the cylindric rotor. After the pre-pressure is adjusted, the set screws placed into the screw holes can prevent relative rotation between the two rotors. The ball bearing is connected to the shaft by interference fit. The base is used to fix the stator. Additionally, the center hole of the shell makes the dual-rotor system and the stator to be coaxial via the ball bearing. The shell is fixed to the base by four screws.

In Figure 1b, the hub is fixed to the cylinder of the base by four screws. The stator consists of the metal ring and 32 pieces of piezoelectric ceramics. The double-sided teeth are staggered on the metal ring, which has the hub and supporting plate. The supporting plate can effectively reduce the bad effect of the fixed hub on the stator vibration. As shown in Figure 1c, all piezoelectric ceramics (PZT) are polarized along the thickness and divided into two groups, PZT-A and PZT-B, according to the location on the metal ring. In PZT-A or PZT-B, polarization directions of two adjacent ceramics are opposite. The staggered configuration of the piezoelectric ceramics and the driving teeth achieves the double-sided driving function of the stator with a compact structure.

The material of PZT-A and PZT-B are PZT-4, and the relevant parameters are listed in Table 1. The material of the metal ring is a hard aluminum alloy (2A12), the density, elastic modulus and Poisson’s ratio of which are 2810 kg/m^3^, 66 GPa and 0.33, respectively.

## 3. Principle of the Stator Vibration

**Figure 2:**
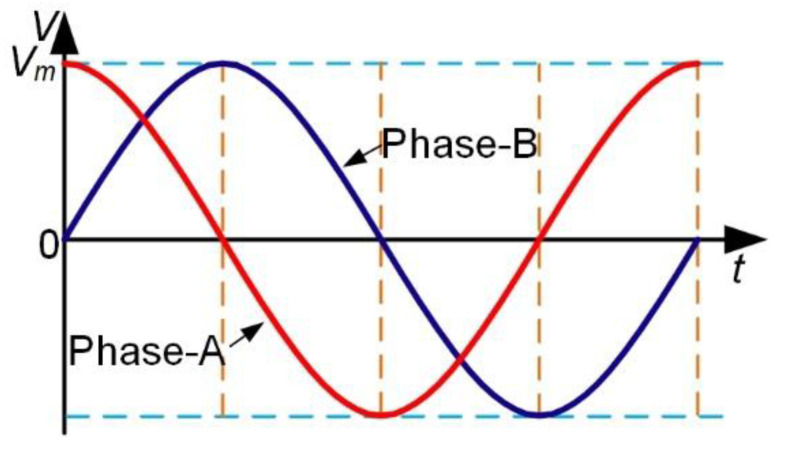
Two sinusoidal alternating voltages with 90° phase difference.

Figure 2 shows two sinusoidal alternating voltages applied to PZT-A and PZT-B respectively, which have a phase difference of 90°. Each group of the piezoelectric ceramics, PZT-A or PZT-B, can generate a standing wave in the stator under the excitation of one alternating voltage.

The following equations represent two standing waves obtained via exciting PZT-A and PZT-B in the stator:

(1) $$\left\{ \begin{array}{l} Y_{1}=\xi \cos kx\cdot \cos\omega t\\ Y_{2}=\xi \cos kx^{\prime }\cdot \cos\omega t^{\prime } \end{array}\right.$$

where *ξ* is the amplitude, *k* is the wave vector (*k = 2π/λ*, *λ* is the wavelength), *x* and *x*′ are space angles, *ω* is the angular frequency of vibration, and *t* and *t*′ are the vibration times. According to the layout of PZT-A and PZT-B, *x*′ = *x*−*λ*/4. Because of the 90° phase difference between two voltages, *t*′ = *t*−*T*/4 (*T* is the cycle). Therefore, the second standing wave equation is also as follows:

(2) $$Y_{2}=\xi \sin kx\cdot \sin\omega t$$

The two standing waves are superposed to form a flexural traveling wave under the above conditions. The traveling wave equation is presented as follows:

(3) $$Y=Y_{1}+Y_{2}=\xi \cos(\omega t-kx)$$

**Figure 3:**
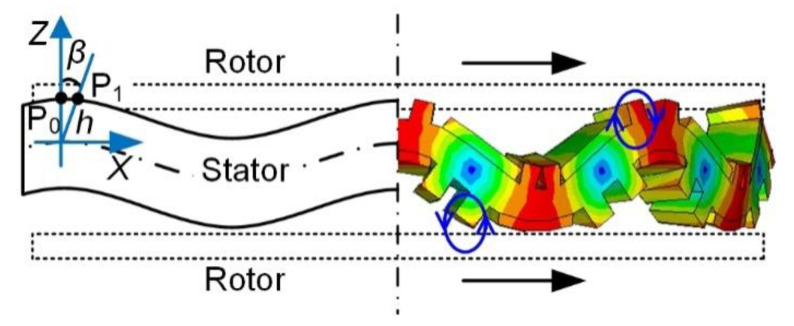
The movement process on the surface of driving teeth.

Figure 3 shows the movement process of any node P on the surface of driving teeth. *h* represents the distance from point P to the neutral surface of the stator. When the traveling wave moves in the stator, the stator ring produces a bending vibration. *β* is the rotation angle of the section where node P is located, and node P moves from point P_0_ to point P_1_.

According to Figure 3, the displacement equations of node P in *Z*-axis and *X*-axis directions could be obtained as follows:

(4) $$\left\{ \begin{array}{l} W_{Z}=Y-h(1-\cos\beta )\\ W_{X}=h\sin\beta \end{array}\right.$$

The bending angle *β* is tiny. Thus, the axial vibration displacement *W_Z_* could be approximated as follows:

(5) $$W_{Z}\approx Y=\xi \cos(\omega t-kx)$$

The beam deforms slightly, so the tangential vibration displacement *W_X_* could be expressed as follows:

(6) $$W_{X}\approx h\beta \approx h\frac{dY}{dx}=h\xi k\sin(\omega t-kx)$$

The relationship between *W_X_* and *W_Z_* satisfies the following equation:

(7) $${\left(\frac{W_{Z}}{\xi }\right)}^{2}+{\left(\frac{W_{X}}{hk\xi }\right)}^{2}=1$$

Therefore, the ellipse trajectories on the surface nodes of driving teeth are formed. In Figure 3, two ellipse trajectories in the top and bottom surfaces of the stator have reverse directions clockwise and anticlockwise, respectively. In this way, the two rotors in contact with the stator rotate in the same direction under the friction force. If the phase difference of two alternating voltages is altered to be −90°, two rotors will rotate in the opposite direction.

## 4. Analysis of the Stator

In order to validate the effectiveness of the proposed motor, the finite element method was adopted to analyze the stator model in this study.

**Figure 4:**
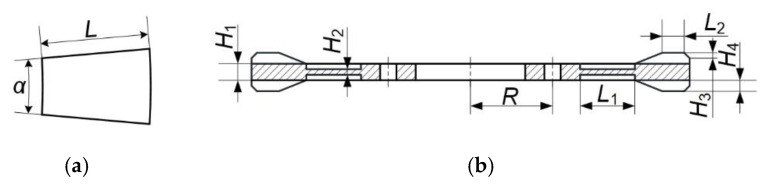
The main structural parameters of the stator: (**a**) Ceramic chip; (**b**) Metal ring.

Figure 4a shows the main structural parameters of the ceramic chip. The thickness, width *L* and central angle *α* of the ceramic chip are 0.5mm, 10mm and 10°, respectively. Figure 4b shows the main structural parameters of the metal ring. *H*_1_ and *R* are the dimensions of the hub, which are determined by the cylinder of the base. Moreover, the hub was fixed during analysis, which might have little effect on the stator vibration. *H*_1_ is also the thickness of the metal ring without regard to the teeth, which determines the axial bending strength of the stator ring. *L*_2_ is the width of the teeth. *H*_4_ is the height of the teeth, which should be a little larger than the thickness of the ceramic chip to prevent contact between the rotor and the ceramics. *H*_2_ is the thickness of the supporting plate to hold the stator ring, which should be thinner than *H*_1_. *L*_1_ is the length of the supporting plate, which is determined by the outer diameter of the stator ring.

**Figure 5:**
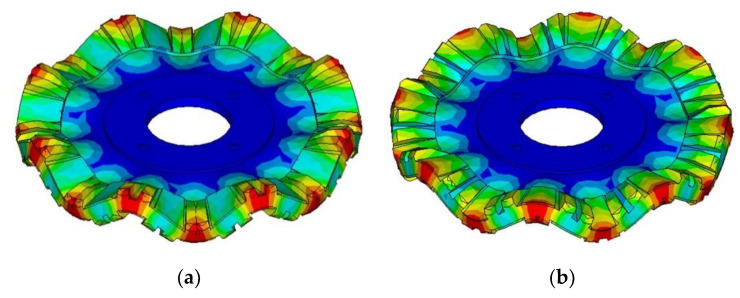
Vibration mode shapes of the stator: (**a**) Mode-A; (**b**) Mode-B.

In this study, the outer diameter of the stator ring is 80 mm, the other dimensions of which are listed in Table 2. Additionally, the model of stator without the hub and the supporting plate was established and analyzed in finite element software firstly. Modal analysis was carried out to extract the resonant frequencies of B(0,8) vibration modes of the stator, which were 29.061 kHz and 29.074 kHz, respectively. Then, the model of the entire stator was established. Figure 5 shows the vibration shapes of the stator, Mode-A and Mode-B, where B(0,8) bending vibration modes can be recognized. B(0,8) bending vibration mode has eight antinodes on either side of the ring. Mode-A is generated by exciting PZT-A on the bottom surface, and Mode-B is generated by exciting PZT-B on the top surface.

The resonance frequencies of the B(0,8) bending vibration mode of the entire stator were 28.992 kHz and 28.998 kHz, respectively, which were 69 Hz and 76 Hz different from that without the supporting plate and the hub. It can be seen that the supporting plate and the hub play an essential role in the vibration characteristics of the stator.

**Figure 6:**
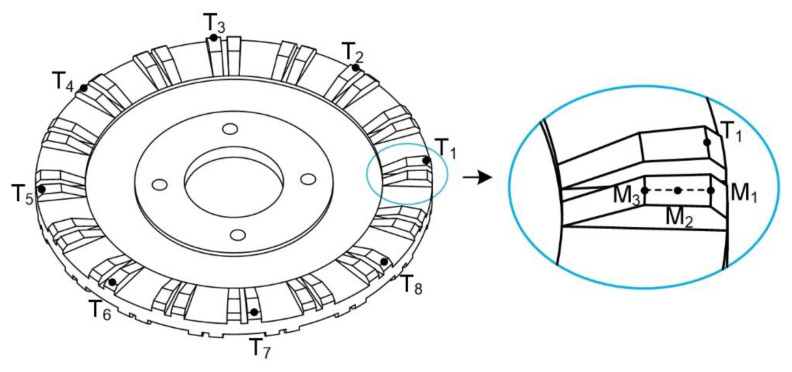
The selection of nodes.

Secondly, transient analysis using the finite element method was analyzed to study the traveling wave in the stator. Additionally, the calculation was carried out under the excitation of two sinusoidal alternating voltages. The frequencies of the two alternating voltages were both 28.995 kHz with the same value of 150 V_p_. Three nodes, M_1_–M_3_, were selected to study the movement of one driving tooth, as shown in Figure 6.

**Figure 7:**
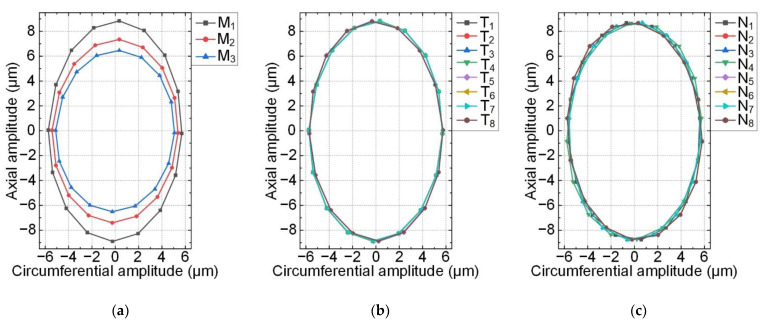
Motion trajectories of nodes: (**a**) three nodes on one tooth; (**b**) eight center nodes on the top surface; (**c**) eight center nodes on the bottom surface.

Figure 7a shows the motion trajectories of three nodes on one driving tooth in one cycle. In Figure 7a, the circumferential vibration displacements of three nodes are approximately equal, but the axial vibration displacements are different. The larger the radius of the node is, the stronger the axial vibration is. Then, eight center nodes of outer edges on eight evenly distributing teeth of the top surface were selected, T_1_–T_8_, as shown in Figure 6. There were another eight nodes selected, N_1_–N_8_, on the bottom surface in the same way. The motion trajectories of the 16 center nodes mentioned above are shown in Figure 7b,c. The motion trajectory consistency indicates that the traveling wave was generated in the stator ring, and rotation directions of the ellipse trajectories on the top and bottom surfaces were opposite. Overall, finite element simulations of the proposed stator model validate that the two rotors could rotate in the same direction.

## 5. Experiments

**Figure 8:**
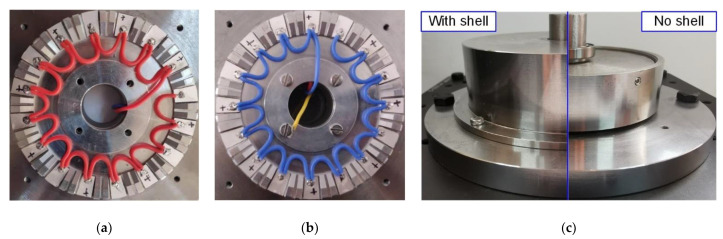
The prototype of the proposed motor: (**a**) the bottom of the stator; (**b**) the top of the stator; (**c**) assembly view with shell and assembly view with no shell.

The metal ring of the stator was machined by a high-precision CNC (computer numerical control) milling machine. Then, the PZT chips were bonded on the metal ring with epoxy resin. Both bottom and top sides of the stator are shown in Figure 8a,b with welded wires. The whole prototype was assembled completely, as shown in Figure 8c.

**Figure 9:**
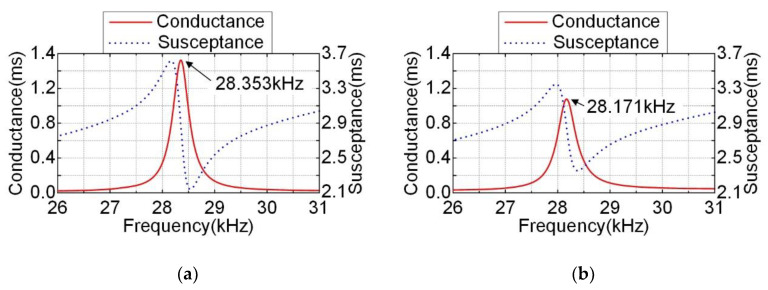
Conductance and susceptance response spectrums under two modes excitations: (**a**) Mode-A; (**b**) Mode-B.

The admittance characteristics of the stator fixed on the base were measured by using an impedance analyzer (E4990A, Keysight Inc, Santa Rosa, CA, USA). Figure 9 shows the admittance test results of the prototype under the excitations of two modes. While PZT-A on the bottom of the stator was connected with the HIGH terminal via the red wire and the metal ring was connected with the LOW terminal via the yellow wire, the test results of Mode-A are shown in Figure 9a. While PZT-B on the top of the stator was connected with the HIGH terminal via the blue wire and the metal ring was connected with the LOW terminal via the yellow wire, the test results of Mode-B are shown in Figure 9b. The resonant frequencies of the two modes were 28.353 kHz and 28.171 kHz, respectively. The admittance test results of the prototype were different from the finite element simulation results, which might result from the manufacturing and assembling process, or neglect of solder joints and epoxide resin, etc.

**Figure 10:**
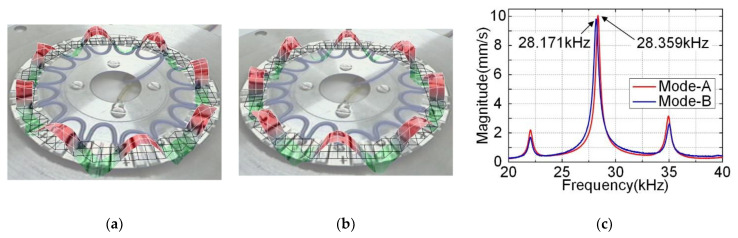
The vibration scanning result of the stator: (**a**) the vibration shape of Mode-A; (**b**) the vibration shape of Mode-B; (**c**) the vibration velocity response spectra under two mode excitations.

Then, the vibration modes of the stator were measured by the use of a scanning laser Doppler vibrometer (PSV-400-M2, Polytec, Waldbronn, Germany). The vibration shapes and vibration velocity response spectrums of the prototype are shown in Figure 10, while wires connection methods were the same as the admittance characteristics test. The top vibration shapes of Mode-A and Mode-B were both standing waves, which have a 90° phase difference in space. The mode shape test results were consistent with Figure 5, but the measured resonant frequencies in Figure 10c were also different from finite element simulation results. However, the resonant frequencies measured in admittance and vibration characteristics tests were almost exactly the same.

**Figure 11:**
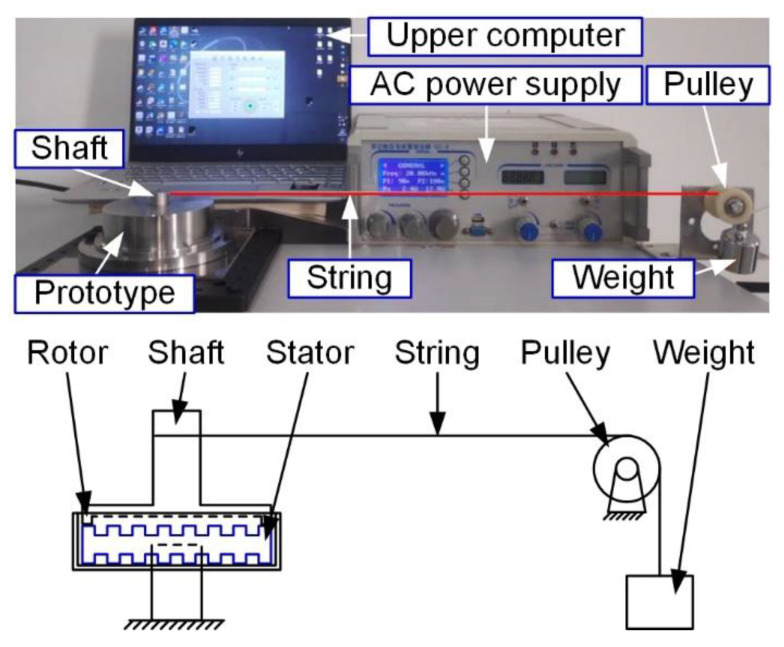
The experiment set-up of the prototype.

The mechanical output performances of the prototype were tested by the experimental set-up, as shown in Figure 11. 

**Figure 12:**
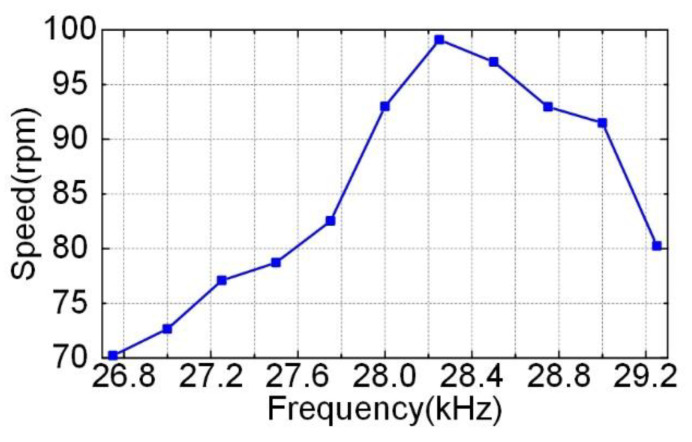
Plot of the speed versus the excitation frequency.

**Figure 13:**
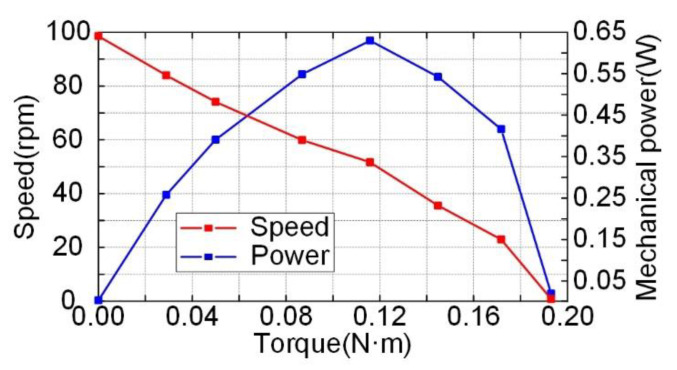
Plot of the speed and power versus the output torque.

If the Phase-A voltage led the Phase-B voltage by 90°, and the dual-rotor system rotated clockwise, then when the Phase-A voltage lagged the Phase-B voltage by 90°, the dual-rotor system rotated counterclockwise. The experiment of the prototype showed that whether the dual-rotor system rotated clockwise or counterclockwise, the test results of mechanical output performance were almost consistent. When the voltages were 150 V_p_, the phase difference was 90° (whether the Phase-A voltage led or lagged the Phase-B voltage by 90°), and Figure 12 shows the plot of the speed versus the frequency of the excitation voltage without the weight loaded. The maximum speed of the prototype was 99 rpm when the excitation frequency was 28.25 kHz. Figure 13 shows the plot of the speed and power versus the output torque when the frequency was set to be 28.25 kHz. With the excitation voltage of 150 V_p_, the motor achieved its maximum torque of 0.19 N·m and a maximum mechanical power of 0.64 W. Compared with Li’s traveling wave ultrasonic motor [27], the weight of the ceramics decreased from 0.039 kg to 0.007 kg, and the load-free speed was increased from 76 rpm to 99 rpm.

## 6. Conclusions

A rotary traveling wave ultrasonic motor with double-sided staggered teeth was proposed. The stator of the motor was constructed by bonding thirty-two pieces of ceramic chips on a metal ring with double-sided staggered teeth. The dual-rotor system was intended to utilize all the traveling wave energies on both sides of the stator, which avoided the energy waste problem of the conventional traveling wave ultrasonic motor. In addition, the corresponding load capacity could be improved efficiently. The modal and transient simulations showed that both sides of the stator could generate the traveling wave to rotate two rotors. The admittance and vibration characteristics tests verified the rationality of the structural design and the simulation results. Additionally, the assembled prototype could achieve double-sided driving stably. Under excitation voltage of 150 V_p_, the prototype achieved a load-free speed of 99 rpm and a maximum output torque of 0.19 N·m. This study provides a new method to achieve double-sided driving of the traveling wave ultrasonic motor.

## Figures and Tables

**Table 1:** The material parameters of PZT-4.

Properties	Values
density (kg/m^3^)	7624.8
piezoelectric stress matrix (C/m^2^)	$\left[\begin{array}{ccc} 0 & 0 & -5.6\\ 0 & 0 & -5.6\\ 0 & 0 & 14.8\\ 0 & 0 & 0\\ 0 & 12.7 & 0\\ 12.7 & 0 & 0 \end{array}\right]$
anisotropic stiffness matrix (10^10^ N/m^2^)	$\left[\begin{array}{cccccc} 14.7 & 8.5 & 7.9 & 0 & 0 & 0\\ 8.5 & 14.7 & 7.9 & 0 & 0 & 0\\ 7.9 & 7.9 & 11.9 & 0 & 0 & 0\\ 0 & 0 & 0 & 3.1 & 0 & 0\\ 0 & 0 & 0 & 0 & 2.6 & 0\\ 0 & 0 & 0 & 0 & 0 & 2.6 \end{array}\right]$
relative permittivity matrix	$\left[\begin{array}{ccc} 762.6 & 0 & 0\\ 0 & 762.6 & 0\\ 0 & 0 & 523.9 \end{array}\right]$

**Table 2:** The dimensions of the stator (unit: mm).

*L*_1_	*L*_2_	*R*	*H*_1_	*H*_2_	*H*_3_	*H*_4_
10	5	15	3	1	1	2

## Data Availability

Not applicable.
